# HDXBoxeR: an R package for statistical analysis and visualization of multiple Hydrogen–Deuterium Exchange Mass-Spectrometry datasets of different protein states

**DOI:** 10.1093/bioinformatics/btae479

**Published:** 2024-07-30

**Authors:** Maria K Janowska, Katherine Reiter, Pearl Magala, Miklos Guttman, Rachel E Klevit

**Affiliations:** Department of Biochemistry, University of Washington, Seattle, WA, 98195, United States; Department of Biochemistry, University of Washington, Seattle, WA, 98195, United States; Lyterian Therapeutics, South San Francisco, CA, 94080, United States; Department of Biochemistry, University of Washington, Seattle, WA, 98195, United States; Department of Medicinal Chemistry, University of Washington, Seattle, WA, 98195, United States; Department of Biochemistry, University of Washington, Seattle, WA, 98195, United States

## Abstract

**Summary:**

Hydrogen–Deuterium Exchange Mass Spectrometry (HDX-MS) is a powerful protein characterization technique that provides insights into protein dynamics and flexibility at the peptide level. However, analyzing HDX-MS data presents a significant challenge due to the wealth of information it generates. Each experiment produces data for hundreds of peptides, often measured in triplicate across multiple time points. Comparisons between different protein states create distinct datasets containing thousands of peptides that require matching, rigorous statistical evaluation, and visualization. Our open-source R package, HDXBoxeR, is a comprehensive tool designed to facilitate statistical analysis and comparison of multiple sets among samples and time points for different protein states, along with data visualization.

**Availability and implementation:**

HDXBoxeR is accessible as the R package (https://cran.r-project.org/web//packages/HDXBoxeR) and GitHub: mkajano/HDXBoxeR.

## 1 Introduction

Hydrogen–Deuterium Exchange Mass Spectrometry (HDX-MS) has emerged as a powerful tool for probing protein structure and dynamics (James *et al.* 2022). Traditional bottom-up HDX-MS experiments monitor the incorporation of deuterium into protein backbone amides over time, resulting in a peptide mass change that is measured via mass spectrometry. The rate of deuterium uptake provides insights into protein structure and dynamics, with more unstructured or dynamic regions exchanging deuterium faster than structured or protected ones. Narrow regions of deuterium incorporation can be identified by using nonspecific proteases into the workflow. While this greatly increases sequence coverage and redundancy, it can also generate hundreds of peptides for subsequent analysis. To ensure data reliability, these measurements are usually repeated (triplicate) and taken across multiple time points to capture the kinetics of deuterium exchange within the protein.

Most biological studies involve perturbation to the system to uncover complex and fundamental mechanisms. Thus, HDX-MS experiments often encompass different protein states, such as the impact of ligand binding or mutations, further increasing the complexity of the HDX-MS experiment and resulting data. Extracting meaningful information from such large datasets requires not only accurate matching of peptides across samples but also rigorous statistical analysis to identify peptides that exhibit significant changes in deuterium uptake between different states.

Analysis of the HDX-MS experiments is time-consuming due to the large amount of data. Furthermore, it requires expertise in statistical and data manipulation that limits approachability and accessibility for researchers whose investigations could otherwise benefit from HDX-MS. Considering the growing use of HDX-MS alongside structural tools such as Cryo-Electron Microscopy and X-ray Crystallography, developing and extending pipelines that can overcome this hurdle is critical to make HDX-MS data analysis accessible to a wider range of researchers.

We have developed HDXBoxeR, a comprehensive R package for the post-processing of HDX-MS data. The package provides a facile analysis-to-publication pipeline that incorporates data input, statistical analysis, dataset comparison, visualization, and preparation of formatted outputs necessary for publication (Hageman and Weis 2019, Masson *et al.* 2019).

HDXBoxeR is streamlined to work with HDExaminer (Trajan Scientific and Medical) output, a processing software that is growing in popularity within the HDX community. The HDExaminer software enables data processing, export functionalities, PyMOL script generation, and visualization tools (see Supplementary Table). Despite these important features, there remains a need for further tools to reduce the complexity of large datasets by focusing on peptides and regions that exhibit significant differences across multiple samples, thereby simplifying the analysis. Thus, we developed HDXBoxeR to streamline HDX-MS data processing and visualization, accommodating multiple protein states, time points, and replicates. Other post-processing softwares that accept modified HDExaminer output include Deuteros (Lau *et al.* 2021) or HaDeX (Puchała *et al.* 2020). The functionalities provided by these and other tools [such as MSTools (Kavan and Man 2011), MEMDX (Hourdel *et al.* 2016), HDX-Viewer (Bouyssié *et al.* 2019)] offer additional and complementary features to HDXBoxeR, but most do not offer customizations for flexible data visualization, curation, and data selection (Kavan and Man 2011, Liu *et al.* 2011, Hourdel *et al.* 2016, Gessner *et al.* 2017, Bouyssié *et al.* 2019, Lumpkin and Komives 2019, Puchała *et al.* 2020, Smit *et al.* 2021, Lau *et al.* 2021, Zhang *et al.* 2021, Quast *et al.* 2022, Seetaloo *et al.* 2022, Cupp-Sutton *et al.* 2023, Largy and Ranz 2023, Uhrik *et al.* 2023, Crook *et al.* 2024). Aforementioned tools can be accessed either via a web server or as a stand-alone programming package (see Supplementary Table).

HDXBoxeR is designed to enable users to compare multiple sets of HDX-MS data, offering flexible tools for quick and comprehensive data analysis. HDXBoxeR uses the Welch’s *T*-test and the critical interval statistical framework to identify statistically significant differences in deuterium uptake between datasets recommended by IC-HDX community discussions (Masson *et al*. 2019). Many of the visualization tools and data exports are already filtered through the lens of significantly different peptides between the protein sets. Additional functionalities include HDX-summary tables, PyMOL script generation (for significantly different peptides between sets), and the creation of various types of plots. HDXBoxeR ensures that generated data adheres to HDX-MS data publication standards and offers an efficient workflow for HDX-MS data analysis and interpretation (Masson *et al.* 2019). In addition, the package can accept input from published datasets, potentially allowing for re-analyses of data collected by different groups.

## 2 Materials and methods

HDXBoxeR provides a comprehensive tool for data reorganization, statistical analysis, and visualization, substantially expediting the differential HDX-MS analysis process. HDXBoxeR, an R package, (Schrödinger 2015, Tellinghuisen and Spiess, 2019 , Neuwirth 2022, R Core Team 2022, Tunisia 2022, Wickham 2023, Wickham and François 2023, Wickham *et al.* 2023) facilitates the processing and comparison of multiple protein states, time points, and replicates. The focus of the package is its ability to compare multiple states (samples) simultaneously. By default, all protein states are compared to the first state within the data, but the order of data analysis or reference data can be changed by the user as desired. We have tested the functionalities using a dataset of a total of eight protein states, four time points, and four replicates simultaneously, but there is no upper limit on the number of states that can be analyzed (Reiter et al., 2022). HDXBoxeR uses a hybrid significance test approach (Hageman and Weis 2019) (the Welch’s *T*-test and the critical interval statistical framework) to assess if the difference in deuterium uptake between sets is statistically significant.

For comprehensive characterization and analysis, HDXBoxeR uses input from HDExaminer (Trajan Scientific and Medical), eliminating the need for additional tools for data preparation, and further facilitating the analysis. The strength of the package lies in its ability to filter multiple protein states through the lens of significantly different peptides.

Although full use of HDXBoxeR requires familiarity with the R programming language, it is designed to allow use by those with only minimal experience with R. Plot generation and visualization do not require writing new functions. More advanced users can leverage the R programming language to extend their analysis, utilizing the prepared intermediate processing files. We highly recommend using our vignette (tutorial) for users to familiarize themselves with the package. The vignette provides a straightforward workflow, enabling users to follow commands to analyze the data. The software can be downloaded from and installed on GitHub: mkajano/HDXBoxeR and is available through CRAN.

### 2.1 Functionality

HDXBoxeR facilitates multiple aspects of the final HDX-MS data analysis. In addition to analyzing multiple protein states, the program has the functionality to:

1. Reprocess input data to the format required for data publication.
2. Calculate parameter tables such as back exchange, average peptide lengths, and statistical information for a general HDX summary table for publication.
3. Convert output from HDExaminer (Trajan Scientific and Medical) to a format that is easier to customize for downstream analysis.
4. Return peptides that are significantly different between sets, using Welch’s *T*-tests.
5. Return scripts for PyMOL to show significantly different peptides between sets and display the HDX results onto a protein structure.
6. Enable rapid plot generation, including:
  - Generic plot generation such as Woods plots, volcano plots, and average deuteration, uptake plots.
  - Welch’s and critical interval statistics filtered plots: heat maps, robot plots (modified butterfly plot), (filtered) Woods plots, and significantly different peptides maps.

**Figure 1.**
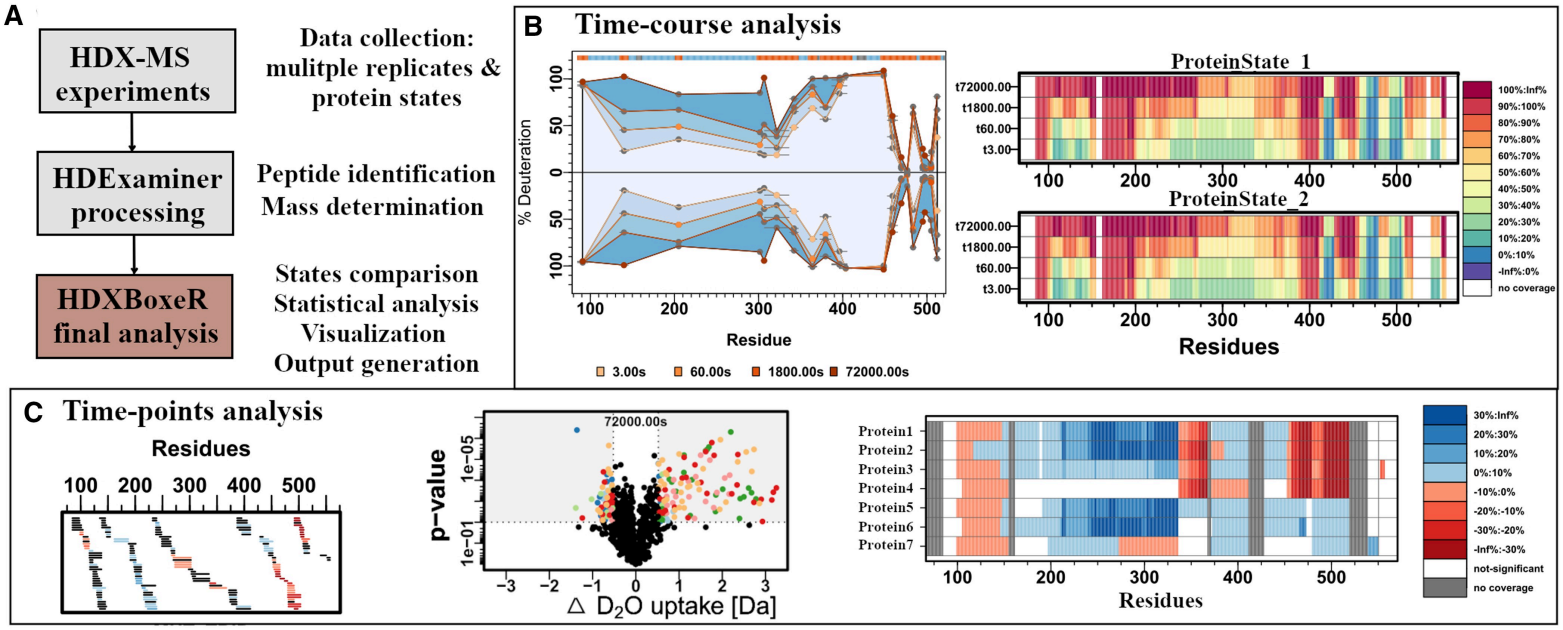
Overview of HDXBoxeR. (A) Scheme showing the pipeline for analysis using the HDXBoxeR R package. (B) Examples of time–course analysis plots generated by HDXBoxeR. Left: Robot plot comparing the percent deuteration of two protein states, only significantly different peptides in at least one time point are shown; Right: Time–course heatmap with legend. (C) Examples of time-point plots generated by HDXBoxeR. Left: Map of significantly different peptides between two states. Middle: Volcano plot. Right: Heatmap comparing deuteration at the same time point for eight protein states (seven states compared to control) protein states based only on significantly different peptides between the states.

HDXBoxeR uses a single input to compare whole datasets and provides statistical analysis for all provided states, time points, and technical replicates. Once the statistical analysis is executed, users can save the data as an output, visualize it, or prepare [filtered through the Welch’s/critical interval statistics (Hageman and Weis 2019)] PyMOL scripts to display the data. HDXBoxeR provides a suite of plots including deuteration difference, volcano plots, heat maps, ‘robot plots’ (modified butterfly plots, highlighting peptide ranges and showing peptides that are significantly different between states), or woods plots (with or without statistics filters) (see Fig. 1) that can be used for data visualization and publication. The suite of plots allows the user to obtain a more comprehensive understanding of the data and facilitates drawing clear conclusions.

HDXBoxeR also has the functionality to return summary information about the sets: time points, number of replicates, number of peptides, peptide coverage, average peptide length, average redundancy, standard deviation, critical interval, average back exchange, and back exchange range. The information provided complies with Masson *et al.* (2019). HDXBoxeR reprocesses HDX-MS data and calculates statistical parameters from it. Data can be exported automatically in different outputs depending on user needs. Users can prepare a format that is ready for publication or return a simple .csv with all the peptides matched among the sets or provide a verbose output that includes peptides' standard deviation and *P*-value per peptide.

The uniqueness of the HDXBoxeR package lies in its ability to compare multiple states within a set. The proposed workflow streamlines the analysis of multiple states and offers various options for easy visualization. Another advantage of the HDXBoxeR package is its strong emphasis on visualizing information involving peptides that display significantly different behavior between protein states.

Novel features in the package include:

- Heatmaps focused only on significantly different peptides between protein states.
- Peptide coverage maps color-coded for significantly different peptides between protein states.
- Two options for Woods plots: one showing all peptides and another focusing only on peptides significantly different between (multiple) protein states [also available through Deuteros (Lau *et al.* 2021)].
- Robot plots highlight significantly different peptides between protein states. These plots are a modification of butterfly plots. Similar to butterfly plots, robot plots are centered around one axis (here the x-axis), but the y-axis is plotted bi-directionally, allowing for a visual comparison of the data. Specifically, in robot plots, the *x*-axis represents residue numbers (unlike in butterfly plots, where it is the peptide index), while the positive and negative *y*-axes represent the percent deuteration. The novelty of the robot plot lies in the explicit drawing of peptide ranges as horizontal bars at *y*-values corresponding to the peptide’s average percent deuteration (similar to woods plots). The standard deviation of the percent deuteration is depicted as a vertical bar. Additionally, robot plots limit the number of peptides plotted by filtering based on *p*-value and critical interval. Robot plots will display specific peptides only if that peptide was determined to be significantly different from the control state at least at one-time point. The time point at which a peptide was significantly different between the states is denoted by a colored dot in the middle of the peptide. Peptides that are not significantly different (in the time series) will have a grey dot at the middle point of the peptide.
- The workflow ensures consistent color schemes across all plots to denote peptides that are significantly different between states, and facilitates exporting PyMOL scripts using the same color scheme.

## 3 Conclusions

HDXBoxeR is a package that provides a complete post-processing workflow and allows for facile and fast data analysis for multiple sets as well as additional functionality to currently available tools selection (Kavan and Man 2011, Liu *et al.* 2011, Hourdel *et al.* 2016, Gessner *et al.* 2017, Bouyssié *et al.* 2019, Lumpkin and Komives 2019, Puchała *et al.* 2020, 2021, Lau *et al.* 2021, Zhang *et al.* 2021, Quast *et al.* 2022, Seetaloo *et al.* 2022, Cupp-Sutton *et al.* 2023, Largy and Ranz 2023, Uhrik *et al.* 2023, Crook *et al.* 2024). Examples of how to use the package can be found in the R vignette that shows examples for the analysis. Our goal is to provide tools for the HDX-MS users to analyze and compare HDX-MS data in a rigorous, statistically sound manner that will allow them to gain additional insights from their highly information-rich data.

## Supplementary Material

btae479_Supplementary_Data

## Data Availability

No new data were generated or analysed in support of this research.
